# Advances in plant proteomics toward improvement of crop productivity and stress resistancex

**DOI:** 10.3389/fpls.2015.00209

**Published:** 2015-04-14

**Authors:** Junjie Hu, Christof Rampitsch, Natalia V. Bykova

**Affiliations:** ^1^Department of Biology, Memorial University of Newfoundland, St. John’sNL, Canada; ^2^Cereal Proteomics, Cereal Research Centre, Agriculture and Agri-Food Canada, MordenMB, Canada

**Keywords:** combinatorial stresses, quantitative techniques, crop productivity, tissue-specific proteomics, subcellular localization

## Abstract

Abiotic and biotic stresses constrain plant growth and development negatively impacting crop production. Plants have developed stress-specific adaptations as well as simultaneous responses to a combination of various abiotic stresses with pathogen infection. The efficiency of stress-induced adaptive responses is dependent on activation of molecular signaling pathways and intracellular networks by modulating expression, or abundance, and/or post-translational modification (PTM) of proteins primarily associated with defense mechanisms. In this review, we summarize and evaluate the contribution of proteomic studies to our understanding of stress response mechanisms in different plant organs and tissues. Advanced quantitative proteomic techniques have improved the coverage of total proteomes and sub-proteomes from small amounts of starting material, and characterized PTMs as well as protein–protein interactions at the cellular level, providing detailed information on organ- and tissue-specific regulatory mechanisms responding to a variety of individual stresses or stress combinations during plant life cycle. In particular, we address the tissue-specific signaling networks localized to various organelles that participate in stress-related physiological plasticity and adaptive mechanisms, such as photosynthetic efficiency, symbiotic nitrogen fixation, plant growth, tolerance and common responses to environmental stresses. We also provide an update on the progress of proteomics with major crop species and discuss the current challenges and limitations inherent to proteomics techniques and data interpretation for non-model organisms. Future directions in proteomics research toward crop improvement are further discussed.

## Introduction

Understanding all levels that regulate adaptive mechanisms and the resilience of crop plants in the context of climate changes is absolutely essential to reach significant achievements in genomics-driven breeding of major crops for high productivity and stress tolerance. A new pattern of frequently occurring extreme weather events has already been taking a toll on agricultural production systems. In addition to increasing amount of genomic information available for both model and non-model plants, the parallel development of bioinformatics techniques and analytical instrumentation makes proteomics an essential approach to reveal major signaling and biochemical pathways underlying plant life cycle, interaction with the environment, and responses to abiotic and biotic stresses. High-throughput proteomic studies have gone beyond simple identification of individual proteins to quantitative profiling, analysis of dynamic post-translational modifications (PTMs), subcellular localization and compartmentalization, protein complexes, signaling pathways, and protein–protein interactions (for reviews refer to Agrawal et al., 2011, 2013; Matros et al., 2011; Pflieger et al., 2011; Hossain et al., 2012).

Growing in the field or cultivated in the laboratory, plant development and productivity are inevitably controlled by various extreme environmental factors such as drought, heat, salinity, cold, or pathogen infection, which may delay or induce seed germination, reduce seedling growth, and decrease crop yields. Proteomics studies can substantially contribute to revealing virtually every aspect of cellular function in plant stress responses, unraveling possible relationships between protein abundance and/or modification and plant stress tolerance. An increasing number of studies have been discussing the contribution of proteomics to deeper insights into the molecular mechanisms of plant responses to stresses and signaling pathways linking changes in protein expression to cellular metabolic events, such as studies using model plants *Arabidopsis* (Wienkoop et al., 2010), rice (see for reviews Singh and Jwa, 2013; Kim et al., 2014) and sorghum (Ngara and Ndimba, 2014). Attributed to the improvement in diverse proteomic technology platforms that combined classical two-dimensional electrophoresis (2-DE) gel-based techniques with mass spectrometry (MS)-based quantitative approaches as well as the accessibility of protein databases of various plant species, major monocotyledonous cereals and dicotyledonous legumes (e.g., maize, wheat, barley, soybeans etc.) have been widely used to study quantitative changes in protein abundance related to different abiotic stresses (Li et al., 2013a; Jacoby et al., 2013a; Deshmukh et al., 2014; Wu et al., 2014a; Kamal et al., 2015).

In the agricultural environment crop plants are subject to a complex set of abiotic and biotic stresses. In addition to studying effects of various stresses applied individually under laboratory controlled conditions, recent evidence shows that simultaneous occurrence of multiple stresses affecting crop growth, yield and physiological traits can cause plants to activate intricate metabolic pathways involved in specific programming of gene expression that uniquely respond to different combinations of stresses (Atkinson and Urwin, 2012). Several different signaling pathways involved in multiple stress-responding mechanisms have been revealed in transcriptome, metabolome, and proteome analysis of various crop plants subjected to different stress combinations, suggesting a complex regulatory network orchestrated by hormone signals, transcription factors, antioxidants, kinase cascades, reactive oxygen species (ROS), and osmolyte synthesis (Suzuki et al., 2014).

Fundamentally, crop growth depends on efficient production of energy and nutritional compounds regulated through different organs, which are equipped with various organelles and organ-specific sets of cytosolic proteins, hormones and metabolites (Komatsu and Hossain, 2013). The responses of plant cells to abiotic stresses vary in different organs. Organ-specific proteomics combined with subcellular organelle proteomic studies of developmental mechanisms from leaf to root can provide more detailed information for understanding of cellular mechanisms that regulate stress response and signal transduction in various organelles (Hossain et al., 2012; Komatsu and Hossain, 2013; **Table 1**). Tissue-targeted seed proteomic studies of different developmental stages under abiotic stresses have contributed to increasing our depth of knowledge about the processes controlling seed development, dormancy and germination by analyzing spatial and functional sub-proteomes (Finnie et al., 2011a). In this article we provide an update on the progress of proteomics with major crop species and discuss the current challenges and limitations inherent to proteomics techniques and data interpretation for non-model organisms.

**Table 1:** Overview of approaches used for subcellular proteomic studies in crop plants under abiotic stress.

Subcellular compartment	Tissue, species	Methodology	Major findings	Reference
Chloroplast	Leaves, *Zea mays*	2-DE, MALDI-TOF MS	20 proteins responded to short-term salt exposure	Zörb et al. (2009)
Chloroplast	Leaves, *T. aestivum*	2-DE, LTQ-FTICR, green plants UniProtKB/Swiss-Prot database	65 proteins responded to salt treatment	Kamal et al. (2012)
Chloroplast	Leaves, *T. aestivum*	1-DE, LTQ-FTICR, green plants UniProtKB/Swiss-Prot database	Thylakoid proteome, identified 767 unique proteins	Kamal et al. (2013)
Chloroplast	Leaves, *Glycine max*	2-DE, MALDI-TOF MS	32 differentially expressed proteins from O_3_-treated chloroplast	Ahsan et al. (2010a)
Mitochondria	Roots, *Oryza sativa*	2-DE MALDI-TOF/TOF MS, PMF and MS/MS analysis	8 program cell death-related proteins	Chen et al. (2009)
Mitochondria	Leaves, *Pisum sativum*	2-DE, nanoLC-MS/MS	26 environmental stress-responsive proteins idenfied	Taylor et al. (2005)
Mitochondria	Shoots, *T. aestivum*	2D-Difference Gel Electrophoresis (DIGE), nanoLC-MS/MS	68 proteins identified; 4 salt stress-responsive	Jacoby et al. (2010)
Mitochondria	Roots and shoots, *T. aestivum, T. aestivum × Lophopyrum elongatum*	2D-DIGE and MALDI TOF/TOF	15 unique salt stress-responsive proteins in shoots, and 33 responsive proteins in roots	Jacoby et al. (2013a)
Cell wall	Root apex, *Zea mays*	2-DE, nanoLC-MS/MS	152 water deficit-responsive proteins	Zhu et al. (2007)
Cell wall	Roots and hypocotyls, *Glycine max*	2-DE, nanoLC-MS/MS, MALDI TOF/MS, Edman degradation	16 out of 204 cell wall proteins were flooding-responsive	Komatsu et al. (2010)
Plasma membrane	Roots and hypocotyls, *Glycine max*	2-DE, MALDI TOF/MS, Edman degradation, gel- and label-free nanoLC-MS/MS analysis	54 and 124 proteins in 2-DE and gel-free, respectively; 22 flooding-responsive proteins	Komatsu et al. (2009)
Plasma membrane	Roots and hypocotyls, *Glycine max*	2-DE, nanoLC-MS/MS, gel- and label-free comparative analysis	osmotic stresss-responsive proteins identified: 12 in 2-DE and 86 in gel-free	Nouri and Komatsu ()
Nucleus	Root tips, *Glycine max*	Gel-free nanoLC-MS/MS, label-free quantitation	39 flooding-responsive proteins	Komatsu et al. (2014)
Plasma membrane	Aleurone, seeds *Hordeum vulgare*	1-DE, nanoLC-MS/MS	46 proteins, over 10 transmembrane domains	Hynek et al. (2006)
Plasma membrane	Embryos, seeds *Hordeum vulgare*	1-DE, nanoLC-MS/MS	61 proteins involved in seed germination	Hynek et al. (2009)

For more comprehensive discussion on previously published organellar and sub-organellar proteomic studies, including model species, refer to Agrawal et al. (2011).

## Approaches and Challenges in Crop Plant Proteomics

With the completion of genome sequences in model species such as dicotyledonous plant *Arabidopsis thaliana*, monocotyledonous crop plant rice (*Oryza sativa* ssp. *japonica* and ssp. *indica*), model grass *Brachypodium distachyon*, legume species soybean (*Glycine max*), *Medicago truncatula* and *Lotus japonicus,* cereal crops *Zea mays,* and *Hordeum vulgare*, other crop species to follow (NCBI list of sequenced plant genomes), and with recently released chromosome-based draft sequence of the hexaploid bread wheat (*Triticum aestivum*) genome (International Wheat Genome Sequencing Consortium [IWGSC], 2014), attention has been focused on linking genomic data and transcriptomic profiles to the spatial and temporal expression, biological function and functional network of proteins. Moreover, dealing with complex and dynamic plant proteomes, it is crucial to choose suitable proteomic approaches targeting the identification of proteins and their modification that may contribute to crop improvement. In the recent years quantitative proteomic studies using high resolution and mass accuracy instruments have been contributing with important information to our understanding of plant growth, development, and interactions with the environment. This capability is especially useful for crops as it may not only contribute to increasing nutritional value and yield, but also to understanding mechanisms of crop adaptation to responses to abiotic stresses.

### Recent Innovative Technologies used in Quantitative Plant Proteomics

Driven by innovations in MS-based technologies and rapid development of quantitative methods, proteomics has emerged as a complementary technique to other approaches such as transcriptomics and metabolomics in the post-genomic era (Wienkoop et al., 2010). Proteomic analysis is achieved through (1) separation and identification of proteins based on 2-DE or coupled gel-free shotgun liquid chromatography tandem mass spectrometry (LC–MS/MS) platforms; (2) elucidation of protein functions and protein functional networks in plant metabolic and signaling pathways through the analysis of protein mapping, characterization of PTMs and protein–protein interactions; (3) bioinformatic strategies and the use of databases for both model and non-model plant species (Holman et al., 2013). Recently, the application of gel-free protein separation approaches and ‘second generation’ proteomic techniques such as multidimensional protein identification technology (MudPIT), quantitative proteomic approaches including isotope-coded affinity tags (ICATs), targeted mass tags (TMTs), and isobaric tags for relative and absolute quantitation (iTRAQ) have been widely used in descriptive and comparative proteomic studies of plant development and metabolic strategies in abiotic stress adaptation. Advances in liquid chromatography-based separation and label-free quantitative proteomic analysis of large number of proteins derived from complex plant samples have recently been discussed (Matros et al., 2011). Although modern gel-free quantitative proteomic approaches, label-based and label-free, are considered to be more advanced and can provide more information on comparative changes in protein expression than one and 2-DE gel-based methods, they have limitations. One obvious limitation in terms of global proteome coverage is the fact that they are designed for less hydrophobic, more aqueous buffer-soluble sub-proteomes, whereas the buffers and detergents used in gel-based protein separation techniques can be quite powerful and efficient in solubilization of more hydrophobic protein groups, especially in conjunction with organellar and sub-organellar pre-fractionation. The development and application of label-free quantitative analysis techniques in combination with one and 2-DE gels was the new realization that helped to solve one of the main drawbacks of gel-based separation and quantitative analysis of proteins related to co-focusing/co-localization of several proteins and their modified forms in one spot or band on the gel.

One of the most socio-economically important agricultural crops is wheat. However, until recently, large-scale proteomic studies with this organism were difficult to realize due to the lack of genomic information available to facilitate protein identifications. The publication of the first draft of completely sequenced wheat genome (International Wheat Genome Sequencing Consortium [IWGSC], 2014) is inspirational to plant proteomics researchers, although the annotation of complete wheat genome assembly will remain a challenging task. Until recently proteomic studies have been using alternative available bioinformatics resources (**Table 2**) that included wheat EST-based databases (Bykova et al., 2011a,b; Pascovici et al., 2013), or available closely related *Brachypodium distachyon* model plant genome, or D-genome progenitor *Aegilops tauschii*, as well as a composite database of available cereals sorghum, maize and rice (Pascovici et al., 2013), a translated database of the low copy number genome assemblies of *T. aestivum* (Alvarez et al., 2014), and proteins from monocot family *Poaceae* (Kang et al., 2015). Pascovici et al. (2013) have evaluated the most effective pipeline for large-scale shotgun quantitative experiments using bread wheat (*T. aestivum*), iTRAQ multirun quantitative approach, and the available resources for bioinformatics data analysis and downstream functional interpretation. The study emphasized many challenges related to the repetitiveness/redundancy/polymorphism of bread wheat genome and therefore extremely large size of the corresponding EST-based database that could not be readily manipulated by the available bioinformatics tools, and the stochastic aspect of protein grouping across multiple runs. The use of smaller databases was demonstrated as alternative pragmatic approaches to reliably identify proteins and proceed with functional annotations (**Table 2**).

**Table 2:** Recent large-scale gel-free quantitative proteomic studies on wheat.

Study aims	Tissue	Quantiative approach	Databases used for protein identifications (IDs)	Number of protein IDs			Reference
				Total (quantified)	Protein groupping	FDR,%	
Evaluate the quantitation pipeline (*T. aestivum*)	leaves, deoxynivalenol treatment	4-plex and 8-plex iTRAQ	1 – NCBInr viridiplantae; 2 – combined cereals; 3 – *Brachypodium*; 4 – *Aegilops tauschii*; 5 – Super Wheat	1 –11884 (1070); 2 – 4770 (1227); 3 – 1808 (985); 4 –1577 (1324); 5 – 54254 (nd)	1162 1262 1023 1353 1694	0.08 0.3 2.3 0.2 nd	Pascovici et al. (2013)
Hydrogen peroxide stress response (*T. aestivum*)	seedlings leaf and coleoptile	4-plex iTRAQ	UniProt: *Triticum*, *Arabidopsis*, *Brachypodium, Oryza*	3425 (157)	1075	≤1	Ge et al. (2013)
Drought response in three different cultivars (*T. aestivum*)	leaves of mature plants	8-plex iTRAQ	Translation of the July 2008 release of the Wheat Gene Index (V11.0,DFCI)	1299 (159)	≥2 peptides, top protein	<1	Ford et al. (2011)
Evaluate drought tolerant and sensitive, role of ABA (*T. aestivum*)	roots	4-plex iTRAQ	Merged: *Triticum* from NCBInr, *Brachypodium* and translated orthogonal assembly of *T. aestivum*	1656 580 836 804	≥2 peptides, top protein	≤1	Alvarez et al. (2014)
Drought response in phosphoproteome, TiO_2_ enrichment (*T. aestivum*)	seedling leaves of two cultivars	Label-free based on precursor ion intensity	Wheat (77,037 entries)	962 (173); 862 (227)	top protein	<1	Zhang et al. (2014b)
Salinity stress and tolerance (*T. durum*)	seedling leaves, control, two salt concentrations	Label-free based on NSAF values	Swiss-Prot viridiplantae	718; 673; 695	≥2 peptides, top protein	≤0.09	Capriotti et al. (2014)
Priming-induced salt tolerance (*T. durum*)	germinating seeds	Label-free, based on normalized spectral counting	Swiss-Prot viridiplantae	380 (182)	≥2 peptides, top protein	≤0.2	Fercha et al. (2013)
Ascorbate priming effect on salt tolerance (*T. durum*)	germinating embryo and surrounding tissues	Label-free, based on normalized spectral counting	Swiss-Prot viridiplantae	697 (167); 471 (69)	≥2 peptides, top protein	≤0.6	Fercha et al. (2014)
Hg-responsive proteins, role of ABA (*T. aestivum*)	seedlings roots and leaves	4-plex iTRAQ	UniProt *Poaceae*	2616 (249)	72% ≥2 peptides, top protein	≤1	Kang et al. (2015)

NSAF, normalized spectral abundance factor; FDR, false discovery rate.

More recently, targeted MS-based quantitative approaches such as multiplexed selective reaction monitoring (SRM) have proven to be powerful for identification of specific proteins with causative functions in agronomically important traits (Jacoby et al., 2013b). Attributable to outstanding sensitivity of this methodology to selective quantitation of low abundance protein components in complex mixtures, SRM technique is seen by researchers as an alternative to antibody-based immunodetection assays (Picotti et al., 2013). This SRM approach is based on highly specific detection and quantitation of proteotypic couples comprised of target precursor and corresponding fragment ions and until recently it was exclusively based on triple quadrupole MS platforms due to the necessity of two stages of mass filters. The advantages and limitations of this application have been discussed and demonstrated experimentally elsewhere. The new generation of Orbitrap-technology instruments such as Q Exactive hybrid quadrupole-Orbitrap provided an efficient and user-friendly alternative for further application of SRM method in quantitative assay development with high potential for large-scale targeted proteomics experiments (Wu et al., 2014b). The purpose of SRM methodology in plant proteomics is biomarker validation in crops, which follows the discovery phase with more explorative qualitative and quantitative comparative proteomic studies aimed at finding potential candidates important in stress responses. This highly selective and sensitive quantitative approach can be powerful not only for biomarker validation but also for the development of new stress tolerance assessment methods, which will facilitate the identification of genotypes with improved resistance and ultimately discovery of gene targets for marker-assisted breeding.

Another approach has recently been developed for label-free shotgun proteomics based on data independent (MS^E^) acquisition protocols that has a potential to identify peptides from complex samples in a rapid, consistent, and sensitive way, and offers a higher dynamic range for peptide quantification (Buts et al., 2014). In this approach, ultra performance liquid chromatography is used, which is coupled to an LC–MS/MS run where an alternating energy level allows to obtain accurate precursor masses at low energy, and to take fragmentation spectra of all parent masses at high collision energy in one analytical run. However, this approach is rather at the early stages of development, and has many challenges related to the difficulties in interpretation of very complex composite fragmentation spectra resulting in poor protein identification rate. At present, parallel data-dependent runs are needed in order to acquire all necessary information, build the databases of individual peptide fragmentation spectra, and link them to the MS^E^ (Buts et al., 2014). This approach was successfully used in quantitative analysis of important allergenic proteins in wheat grain extracts, in identification of gliadins and glutenins in wheat grain and quantitation of proteins associated with celiac disease and baker’s asthma (Uvackova et al., 2013a,b).

An alternative strategy that combines high specificity data-independent acquisition method with a novel targeted data extraction approach to mine the resulting fragment ion data sets was recently demonstrated (Gillet et al., 2012). This method, termed SWATH MS, is based on sequential time-and mass-segmented acquisition, which generates fragment ion spectra of all precursors in two user-defined dimensions, retention time and *m/z* space, resulting in complex fragment ion maps. The interpretation of highly specific multiplexed data sets required the development of fundamentally different data analysis strategy, which uses previously acquired information contained in spectral libraries to mine the fragment ion maps for targeted extraction and quantitation of specific peptides of interest. The accuracy and consistency of SWATH MS was demonstrated to be comparable to SRM approach (Gillet et al., 2012). One of the important advantages of the former, alleviating most constrains of present proteomics methods, is the iterative retrospective re-mining of the acquired data sets for targeted extraction. This approach offers unprecedented possibilities for the qualitative and quantitative profiling not only in proteomics but also in metabolomics and lipidomics. One of the main bottlenecks for proteomics development is the lack of robust bioinformatics tools with novel algorithmic solutions to processing of MS data, which are lagging behind the substantial advances occurring in instrumentation and protocols (Cappadona et al., 2012; Smith et al., 2014). It remains to be seen how this breakthrough technology will evolve into a powerful tool utilized throughout plant sciences.

### From Cellular Proteome to Subcellular Protein catalogs

The major advances in organelle-based proteomics have not only provided a deeper insight into protein localization and organelle-specific function in plant biological processes, but also a better understanding of the functions of organelles in metabolic processes involved in plant development and growth (for a recent review refer to Agrawal et al., 2011). The accurate description of an organelle proteome requires the ability to purify organelles from cellular mixture and identify low abundance proteome extracted from these organelles. Several quantitative proteomic studies have focused on spatial subcellular proteomes with particular methods designed for different organelles (references in **Table 1**).

Proteomic analysis of wheat chloroplasts using a combination of two complementary approaches Tricine SDS–PAGE and 2-DE coupled to a high throughput MS methods (LTQ-FTICR and MALDI-TOF/TOF) has contributed to a better understanding of the responsive proteins in photosynthesis during abiotic stress in plastids (Kamal et al., 2012, 2013). Chloroplast proteomics study of soybean leaves under ozone stress by 2-DE and MALDI-TOF MS approach identified increased expression level of proteins involved in antioxidant defense and carbon metabolism (Ahsan et al., 2010a). Proteomic studies on mitochondrial organelles provided information on both individual proteins and protein complexes that participate in salinity response mechanisms in rice through 2-DE and MALDI-TOF MS (Chen et al., 2009), and in wheat through 2-DE and LC–MS/MS analysis (Jacoby et al., 2010, 2013a). Moreover, the effects of drought, cold and herbicide treatments on mitochondrial proteomics in pea were also analyzed by 2-DE Blue Native PAGE with Q-TOF MS (Taylor et al., 2005). Cell wall proteomic studies of major crops such as rice (using 2-DE and nanoLC–MS/MS), soybean (using 2-DE, MALDI-TOF MS, and nanoLC–MS/MS), and maize (using 2-DE and Q-TOF MS), provided insights into either dehydration- or water stress-responsive proteomes involved in a variety of functions, including carbohydrate metabolism, cellular defense through redox mechanisms, cell wall modification, and cell signaling pathways (Zhu et al., 2007; Komatsu et al., 2010). Plasma membrane, as a primary interface between the cellular cytoplasm and the extracellular environment, plays a vital role in signaling, communication and uptake of nutrients (Alexandersson et al., 2004). Due to their low abundance and low solubility, nanoLC–MS/MS-based proteomic approaches have been used to study osmotic stress-induced proteins in soybean, which are mostly involved in antioxidative defense system (Komatsu et al., 2009; Nouri and Komatsu, 2010). Changes in nuclear proteins under the flooding stress in soybean root tips, studied by gel-free nano-LC MS/MS, revealed differentially regulated responding proteins (Komatsu et al., 2014). Moreover, reversed-phase chromatography, SDS–PAGE and LC–MS/MS have been used to prepare and analyze integral plasma membrane-enriched tissue fractions from barley aleurone layers and germinated embryos, providing more information about aleurone layer as a secretory tissue during seed germination (Hynek et al., 2006, 2009). The accurate description of an organelle proteome requires the ability to identify genuine protein residents (**Table 1**). Although many challenges remain, quantitative proteomic profiling of organelles has been developed to reliably identify the protein complement of whole organelles, as well as for protein assignment to subcellular location and relative protein quantification, which are improving our understanding of protein functions and dynamics in plant cells.

## Organ-Specific Proteome Analysis of Abiotic Stress Responses in Crop Plants

### Proteomics of Leaf Photosynthesis and Senescence to Understand Crop Productivity

**FIGURE 1:**
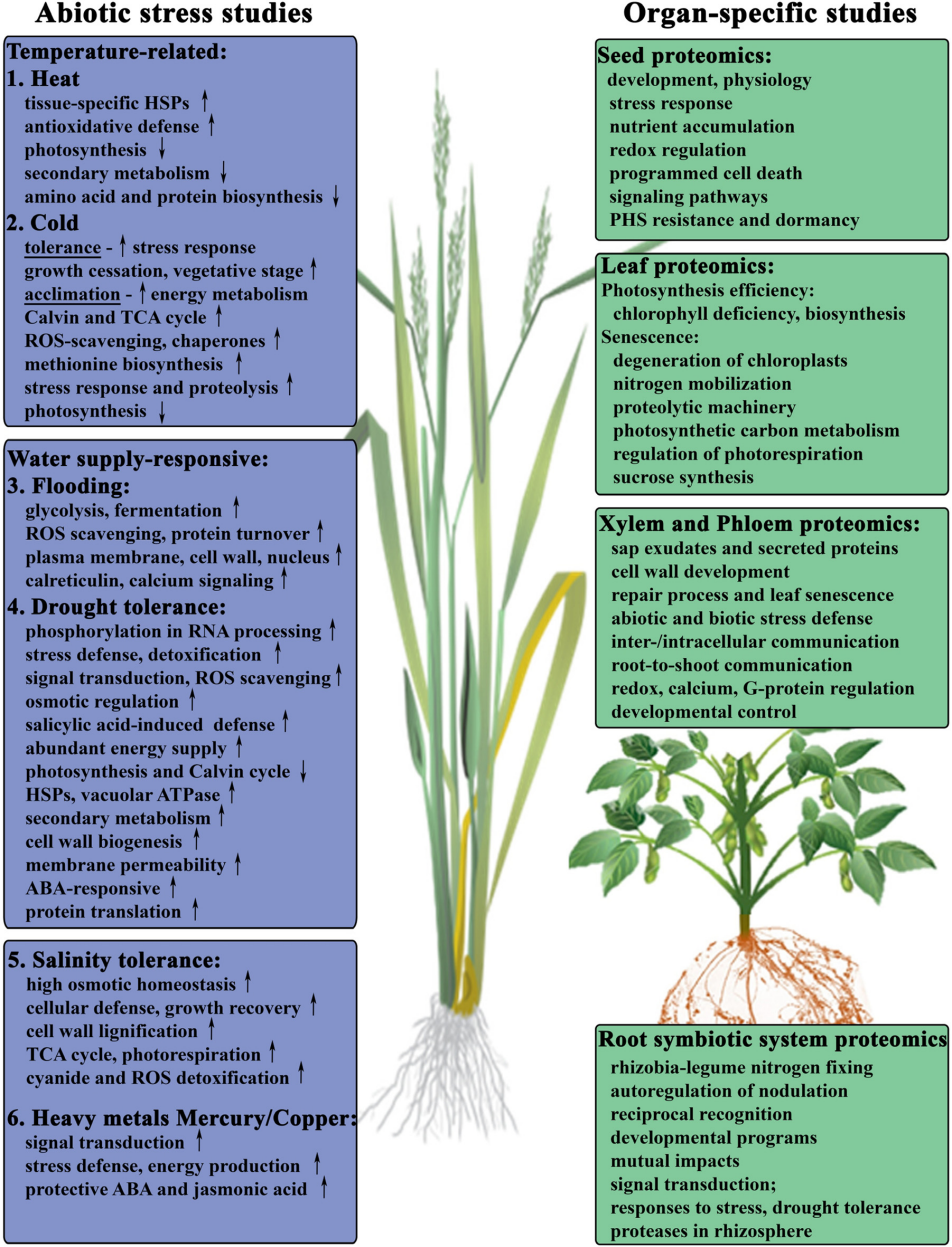
**Proteomic studies-driven insights into cellular activities during abiotic stress response of major crops.** Summary of the abiotic stress studies published in: (1) Heat in soybean (Ahsan et al., 2010b). (2) Cold in winter wheat (Vítámvás et al., 2012; Kosová et al., 2013). (3) Flooding and anoxia in soybean seedlings (Nanjo et al., 2011 review; Komatsu et al., 2009, 2010; Yin et al., 2014). (4) Drought tolerance in wheat roots (Alvarez et al., 2014) and leaves (Ford et al., 2011; Kang et al., 2012; Zhang et al., 2014b). (5) Salinity in wheat (Guo et al., 2012; Jacoby et al., 2013a; Capriotti et al., 2014; Fercha et al., 2014), soybean (Ma et al., 2012), rice (Ruan et al., 2011; Song et al., 2011; Liu et al., 2013b), and barley (Witzel et al., 2009; Gao et al., 2013). (6) Metal ions stress in wheat (Li et al., 2013b; Kang et al., 2015).

Leaf photosynthesis is the main source of plant biomass influencing potential crop yield. Highly abundant chlorophyll in leaves plays essential roles in light harvesting and energy transfer during photosynthesis, therefore, chlorophyll metabolism contributes to photosynthetic efficiency during leaf development. Recently, Chu et al. (2015) analyzed changes in protein profiles upon the development of chlorophyll deficiency in *Brassica napus* leaves and provided new insights into the regulation of chlorophyll biosynthesis and photosynthesis in crops (Chu et al., 2015). Moreover, the levels of chlorophylls were also shown to be associated with the maintenance of photosynthetic rate of CO_2_ consumption during the grain-filling period, and with the rate of leaf senescence in different rice cultivars (Panda and Sarkar, 2013). Leaf senescence is featured with loss of photosynthetic activities and hydrolysis of macromolecules followed by the degeneration of chloroplasts and remobilization of the hydrolyzed nutrients to young leaves and developing seeds (Guo, 2013). Several studies have focused on the proteomics of leaf senescence, mainly on the investigation of nitrogen mobilization from leaves during leaf senescence (Bazargani et al., 2011; Avice and Etienne, 2014). The results of these studies suggest the importance of proteolysis, chloroplast degradation and nitrogen remobilization during this process (**Figure 1**). Chloroplast contains up to 75% of leaf nitrogen in the form of Rubisco enzyme components in the stroma and complex of photosystem II in the thylakoid membrane (Roberts et al., 2012). Advances in organelle proteomic studies integrated with large-scale genomic approaches and determination of enzymes with proteolytic activity have addressed the complexity of chloroplastic proteolytic machinery during leaf senescence and investigated different classes of senescence-associated proteases with unique physiological roles according to their expression profiles along the senescence progress (Liu et al., 2008; Roberts et al., 2012). Moreover, the regulation of photosynthetic carbon metabolism has also been investigated during leaf senescence in rice by a comparative proteomic approach, contributing to a deeper insight into the enzymatic regulation involved in the Calvin cycle during senescence featured with down-regulated photorespiration (Zhang et al., 2010). Furthermore, sucrose as the main photosynthetic product is rich with carbon and energy, is a key component in carbon metabolism, and is essential for both plant growth and the synthesis of storage reserves, such as starch and oil (Barsan et al., 2012). Glycolytic enzymes involved in sucrose synthesis are of particular interest with respect to crop yield, and have been identified by subcellular proteomic studies of senescence, photosynthesis, and stress-responding processes in rice leaves (Zhang et al., 2010).

### Xylem and Phloem Proteomics of Root-to-Leaf Signaling Pathways During Stress

Maximizing crop yields also depends on the leaves receiving an optimal supply of nutrients from the root system via the xylem vessels. The xylem sap is a dynamic fluid circulated between root and leaf, featured with a high content of secreted proteins undergoing changes in its proteome upon abiotic and biotic stresses (Ligat et al., 2011; de Bernonville et al., 2014; Zhang et al., 2014a). Xylem proteomic and secretomic studies have recently become one of the major areas of interest in understanding plant development and responses to environmental perturbations, and illustrated several types of xylem sap-containing proteins that participate in cell wall development and repair process (Kalluri et al., 2009; Ligat et al., 2011; Zhang et al., 2014a), leaf senescence (Wang et al., 2012), abiotic stress responses (Alvarez et al., 2008), biotic stress defense mechanisms (González et al., 2012), and intercellular and intracellular communication (Agrawal et al., 2010). Additional studies of protein and metabolite composition of xylem sap and apoplast in soybean (*Glycine max*) provide further investigation of expression profiles and signaling roles of corresponding proteomes, and ultimately reveal more root contributions to pathogenic and symbiotic microbe interactions, and root-to-shoot communication (Djordjevic et al., 2007; Subramanian et al., 2009; Krishnan et al., 2011).

The phloem tissue isolated from broccoli (*Brassica oleracea*) was found to be enriched in proteins associated with biotic and abiotic stress responses and structural proteins (Anstead et al., 2013). Other proteomic studies were predominantly focused on the analysis of phloem sap exudates from agriculturally important plants oilseed rape (Giavalisco et al., 2006), rice (Aki et al., 2008), pumpkin (Lin et al., 2009; Cho et al., 2010; Fröhlich et al., 2012), and melon (Malter and Wolf, 2011), identifying several hundred physiologically relevant proteins and ribonucleoprotein complexes. The phloem sap proteomes showed enhanced presence of proteins involved in redox regulation, defense and stress responses, calcium regulation, RNA metabolism and G-protein signaling (**Figure 1**). Some of the important insights into the operation of the sieve tube system were revealed through proteomics studies. The findings indicate likely occurrence of protein synthesis and turnover within the angiosperm phloem translocation stream, processes that were thought to be absent in enucleate sieve elements (Lin et al., 2009). There is also indication that phloem may exert some level of control over flowering time (Giavalisco et al., 2006), seed development (Lin et al., 2009), other developmental processes via gibberellin biosynthesis or modification (Cho et al., 2010).

### Root Proteomics of Symbiotic Systems to Improve Legume Productivity

In associations between plants and soil microorganisms, only symbiotic interactions have beneficial effects for the host plants while pathogenic interactions lead to severe damages (Schenkluhn et al., 2010). Rhizobia-legume symbiosis is the best characterized beneficial plant–bacterial mutualistic interaction, which represents one of the most productive nitrogen-fixing systems and effectively renders legumes being independent of other nitrogen sources (Mathesius, 2009; Colditz and Braun, 2010). Legume plants maintain control of nodulation to balance the nitrogen gains with their energy needs and developmental costs through a systemic mechanism known as the autoregulation of nodulation that involves peptide hormones, receptor kinases, and small metabolites for long-distance signaling control (Reid et al., 2011, 2012). Legumes are among the most economically important crops due to their high protein content. Model legume plant *M. truncatula* is closely related to major legume crops such as soybean (*Glycine max*) and pea (*Pisum sativum*), therefore its investigation is of high relevance for agriculture. Recently, comparative and quantitative proteomic studies provided in-depth characterizations of *M. truncatula* proteome focused on symbiosis- or pathogenesis-induced changes of root system (Schenkluhn et al., 2010; Molesini et al., 2013). Moreover, the establishment and maintenance of rhizobium–legume symbiosis require reciprocal recognition with exchanges of signal molecules and complex developmental programs between the organisms, which leads to the formation of nodules on the legume root and the differentiation of rhizobial cells into bacteroids (del Giudice et al., 2011). A number of proteomic studies have attempted to investigate mutual impacts between symbiotic or pathogenic bacteria and the root of host plant in the rhizosphere under a multitude of biotic and abiotic stresses from the soil (for a recent review refer to Knief et al., 2011). Especially under drought stress, symbiotic nitrogen fixation is one of the physiological processes to first show stress responses in nodulated legumes (**Figure 1**). Differential plant and bacteroid responses to drought stress have been revealed by proteomic analysis of root nodule in *M. truncatula* (Larrainzar et al., 2007). Large-scale phosphoproteome analyses focused on nitrogen-fixing root nodules in *M. truncatula* (Wienkoop et al., 2008; Grimsrud et al., 2010) and root hairs in soybean (Nguyen et al., 2012) investigated phosphorylation-mediated signal transduction cascades. Symbiotic signals produced by the rhizobia during the initiation of symbiosis and the development of nodules were revealed, suggesting a complex network of kinase-substrate and phosphatase-substrate interactions in response to rhizobial infections. Furthermore, improved knowledge of root proteases associated with rhizosphere and drought tolerance reinforces the importance of their role in endocytosis of proteins, peptides and microbes, or root exudate-mediated nitrogen uptake mechanisms, which can contribute to a systematic study of root proteases in crop improvement (Adamczyk et al., 2010; Kohli et al., 2012). Additionally, numerous studies have shown that root sensing of stresses from soil drying and salinization can regulate chemical root-to-leaf signaling through altering the complexity of constituents and their interactions in xylem sap, which ultimately reduce leaf transpiration and growth, and therefore, influence crop productivity (Bazargani et al., 2011; Martin-Vertedor and Dodd, 2011).

## Proteomic Studies of the Interaction Between Seed Viability, Seedling Growth and Abiotic Stress

### Tissue-Specific Proteomic Studies of Seed Developmental Stages During Abiotic Stresses

Seed developmental events are programmed to occur as a result of expression and activation of different proteins in distinct seed compartments (i.e., embryo, endosperm, and caryopsis coat) and even within specific regions (e.g., apical meristem), at distinct developmental stages (Itoh et al., 2005). Tissue- and organelle-specific proteomic studies relevant to seed development focused on characterization of temporal and spatial proteomes together with PTMs in metabolic and molecular events that occur at different seed developmental stages and the transit phases between stages (for review refer to Finnie et al., 2011a). These studies provided an insight into the physiological and biochemical pathways, stress-responsive mechanisms, nutrient accumulation and its regulation, redox regulation in programmed cell death, ferredoxin/thioredoxin-linked metabolic processes and signaling pathways, which are specific to embryo (Huang et al., 2012; Xu et al., 2012; Domínguez and Cejudo, 2014; Wolny et al., 2014), aleurone layer (Hägglund et al., 2010; Finnie et al., 2011b; Barba-Espín et al., 2014), endosperm (Vensel et al., 2005; Balmer et al., 2006), and peripheral layers (Jerkovic et al., 2010; Tasleem-Tahir et al., 2011; Miernyk and Johnston, 2013), and whole kernel solubility-based protein groups (Yang et al., 2011). These proteomic studies elucidated the molecular pathways underlying the control of seed development and physiological transitions, which contribute to the advancement of valuable and potentially agriculturally important strategies for improving yield, quality, and stress tolerance in cereals and legumes.

Pre-harvest sprouting (PHS) causes substantial losses in grain yield and quality, and therefore, it is one of the major factors negatively affecting the quality of crops in the areas with high levels of precipitation during grain maturation. Cereal crops with low levels of seed dormancy are susceptible to PHS when wet and moist conditions occur prior to harvest. A defined level of seed dormancy is under genetic, epigenetic regulation and environmental control, and it is an essential component of seed quality (Graeber et al., 2012). PHS resistance is a complex trait that is determined by genotype together with a number of other factors: stage of maturity, environmental conditions during grain ripening, crop morphology, biotic and abiotic stress (Fofana et al., 2008; Rikiishi and Maekawa, 2014). Proteomics offers the opportunity to examine simultaneous changes and to characterize temporal patterns of protein accumulation occurring during seed dormancy maintenance or release (Rajjou et al., 2011). Given that PHS is closely associated with seed dormancy, it is important to gain a deeper understanding of biomarker proteins and molecular signaling mechanisms involved in dormancy regulation, which will contribute to the breeding of PHS resistant cultivars during crop production. Recent proteomics and transcriptomics studies have indicated that antioxidant defense mechanisms, redox regulation of seed mRNAs and protein thiols, integrated with hormonal signaling play a key role in controlling seed dormancy in wheat (Bykova et al., 2011a,b; Liu et al., 2013a), barley (Bahin et al., 2011; Ishibashi et al., 2012), and rice (Huh et al., 2013; Liu et al., 2014). These findings will significantly contribute to the development of efficient strategies for breeding of PHS tolerant crops.

### Proteomic Analysis of Crop Seedlings Subjected to Abiotic Stress Conditions

Seeds break dormancy and restart their metabolism to prepare for germination then proceed with seedling establishment when environmental conditions are suitable for seedling growth and development (Arc et al., 2011). However, crop seedlings in their early growth stage are subjected to various abiotic stresses in the field, which will lead to lower yields and possible crop failure. Quantitative proteomic analysis of soybean seedlings and wheat roots subjected to the flooding or osmotic stresses has revealed the metabolic pathways of flooding-responsive proteome reacting to excess water supply and anoxia, while osmosis-related proteins were responding to the various stresses such as drought, cold, and salinity stresses (Nanjo et al., 2011 and references therein). The initial changes in soybean root tip proteome under flooding stress indicated an important role of calcium signaling in the early responses (Yin et al., 2014), and the exogenous calcium treatment was shown to have a recovery effect on the expression of proteins involved in cell wall, hormone metabolisms, protein degradation and synthesis, and DNA synthesis in soybean roots under flooding stress (Oh et al., 2014). A comparative proteomic study of tissue-specific proteome in soybean seedlings under heat stress indicated common defense and adaptive mechanisms associated with elevated induction of several tissue-specific heat shock proteins and proteins involved in antioxidative defense (**Figure 1**). The down-regulated proteins were associated with photosynthesis, secondary metabolism, and amino acid and protein biosynthesis in response to heat stress (Ahsan et al., 2010b). Salinity stress-responsive proteins in seedlings have been investigated through a comparative proteomic analysis of salt-tolerant and salt-sensitive varieties of wheat (Guo et al., 2012) and soybean (Ma et al., 2012). A significant number of salt tolerance-related proteins were identified in wheat seedling roots, including signal transduction-associated proteins, carbon, amino acid and nitrogen metabolism proteins, detoxification and defense-related proteins (Guo et al., 2012). In a recent proteomics study of soybean seedling leaves, a salt stress-responsive protein network was proposed, including proteins responsible for redox homeostasis, as well as accelerated proteolysis, which was accompanied by reduced biosynthesis of proteins, impaired photosynthesis and energy supply, and enhanced ethylene biosynthesis (Ma et al., 2012). Recently, a comparative label-free shotgun proteomic analysis on wheat leaves of salt-stress tolerant genotype of durum wheat (*T. durum* Desf.; **Table 2**) subjected to increasing salinity levels revealed major changes in proteins involved in primary metabolism, energy production, protein metabolism and cellular defense, as well as tolerance-related high capacity for osmotic homeostasis, and increased cell wall lignification allowing for higher growth recovery potential (Capriotti et al., 2014). In order to understand the role of ascorbate priming in boosting the salt stress tolerance during germination and early seedling growth in durum wheat, a label-free quantitative analysis of whole seeds (Fercha et al., 2013), as well as seed embryos and surrounding tissues was performed to study tissue-specific variation of metabolic proteomes (Fercha et al., 2014). It was demonstrated that ascorbate pretreatment prevents the effect of salinity by specifically changing the abundance of proteins involved in metabolism, protein destination and storage categories, which could be modulated by methionine, auxin and other hormones metabolism, as well as ROS managing and signaling systems (Fercha et al., 2014). These studies provided insights into possible management strategy of cellular activities occurring in salt-responding seedlings of major crops (**Figure 1**).

Among the cereals, rice (*Oryza sativa* L.) is a salt-sensitive crop. High salinity causes delayed seed germination, slow seedling growth, and reduced rate of seed maturation, leading to decreased rice yield. In the early period of growth, rice seedling roots, leaf sheath, and leaf blade are highly sensitive to salt stress signal, which is perceived via plasma membrane receptors and can rapidly initiate an intracellular signal to elicit an adaptive response (Ruan et al., 2011). Abbasi and Komatsu (2004) conducted a proteomic analysis of rice leaf sheath, leaf blades, and roots to investigate relative abundance of salt-responsive proteins, which changed according to intracellular ion homeostasis caused by continuous excessive ion uptake. In another study by Yan et al. (2005), it was suggested that energy production was activated in rice roots subjected to salt stress due to the disruption of enzyme activities and basic metabolism. Kim et al. (2005) elucidated differentially expressed proteins in rice seedling leaves during salt stress as being functionally important in major photosynthetic metabolic processes and in oxidative damage processes. Another study supported findings of the relationship between enzymes involved in carbohydrate and energy metabolism and the increased production of antioxidants that mediate maintenance of cellular homeostasis (Liu et al., 2013b). Song et al. (2011) predicted an extracellular salt stress-responsive apoplastic protein network in shoot stems of rice seedlings, suggesting candidate proteins involved in initial perception of salt stress, such as enzymes involved in carbohydrate metabolism, the intracellular equilibrium between ROS production and ROS scavenging, and protein processing and degradation. Moreover, transgenic rice seedlings overexpressing the cyclophilin OsCYP2 showed improved tolerance to salt stress, with an increased antioxidant enzyme activity and a decreased lipid peroxidation, which indicated a role for OsCYP2 in controlling oxidative damages by modulating activities of antioxidant enzymes at translational level (Ruan et al., 2011). Barley (*Hordeum vulgare* L.), on the other hand, is a salt-tolerant cereal crop and shows variation for tolerance toward salinity stress. MS-based proteomic analysis revealed cultivar-specific and salt stress-responsive protein expression patterns, indicating that proteins involved in the glutathione-based detoxification of ROS were highly expressed in the salt-tolerant genotype, while proteins involved in iron uptake were abundantly expressed in the salt-sensitive genotype. This study emphasized the role of proteins involved in ROS detoxification during salinity stress, and identified potential candidates for increasing salt tolerance in barley (Witzel et al., 2009). Moreover, a comparative transcriptional profiling of barley seedlings under salt stress elucidated a large number of hormone-related kinases and protein phosphatases involved in defense responses against salinity (Gao et al., 2013). These findings provide important information that will be used toward improving salt tolerance of cereals.

More recent proteomics research on the effect of environmental changes and abiotic stress in wheat can be grouped into studies focused on either water supply-responsive proteome reacting to drought (Bazargani et al., 2011; Ford et al., 2011; Ge et al., 2012; Kang et al., 2012; Budak et al., 2013; Alvarez et al., 2014; Zhang et al., 2014b), or temperature-related changes from extreme heat (Yang et al., 2011) to cold (Rinalducci et al., 2011a,b; Kosová et al., 2013) and frost tolerance (Vítámvás et al., 2012; Han et al., 2013), or salinity tolerance and stress (Kamal et al., 2012; Guo et al., 2012; Fercha et al., 2013, 2014; Jacoby et al., 2013a; Capriotti et al., 2014), and heavy metal toxicity stress (Li et al., 2013b; Kang et al., 2015). Availability of moisture during early growing season is critical to wheat production and prolonged drought conditions due to climate change have major impact on the crop yield. Using large-scale isobaric tags-based shotgun quantitative approach, differences in drought stress-responsive proteomes were studied in leaves from mature plants of three wheat varieties, fast and slow responding drought tolerant and intolerant (Ford et al., 2011; **Table 2**; **Figure 1**). An increase in proteins involved in ROS scavenging and a down regulation of proteins involved in photosynthesis and the Calvin cycle were observed in cellular response of all three varieties with the tolerant variety showing significant protein changes during early response and increases in proteins involved in cell detoxification. In a recent study Alvarez et al. (2014) used two wheat varieties adapted to different environmental conditions, drought tolerant and sensitive, to quantitatively evaluate inherent differences in protein expression patterns overall and in variety-specific effect of abscisic acid (ABA) on the root proteome (**Table 2**; **Figure 1**). It was revealed that the tolerant wheat variety had significantly higher number of ABA-responsive and ABA-induced proteins, most of those in the categories representing response to environmental stress and oxidation-reduction processes, which can play an important role in drought adaptation. Another study showed the effect of salicylic acid treatment on the induction of drought tolerance in wheat seedlings and identified salicylic acid-responsive protein interaction network suggesting more effective defense systems, efficient photosynthesis, active anabolism, and abundant energy supply (Kang et al., 2012). Significant changes in the phosphoproteome under drought stress were demonstrated in seedling leaves of two bread wheat cultivars using gel-free TiO_2_ phosphopeptide enrichment and label-free relative quantiative approach (Zhang et al., 2014b). Those mainly concerned proteins involved in RNA transcription/processing, stress/detoxification/defense, and signal transduction, drought tolerance and osmotic regulation.

Two wheat cultivars of contrasting temperature growth habits, frost tolerant winter wheat and frost sensitive spring wheat, were used recently to study cold-induced changes in the proteomes of crown tissues (Kosová et al., 2013). Proteins involved in the regulation of stress response, growth cessation and maintenance of vegetative stage were specifically increased in the winter cultivar, whereas proteins involved in restoration of cell division, plant growth and development, and progression to reproductive stage were induced by cold treatment in the spring cultivar. In an earlier study proteomic analysis of the changes induced by a prolonged treatment of vernalization-requiring winter wheat with low temperatures demonstrated down-regulation of several photosynthesis-related proteins and a concomitant increase in abundance of some Calvin and TCA cycle enzymes, and proteolysis (Rinalducci et al., 2011a). Another study on the effect of long-term cold acclimation and dynamics of cold tolerance on crown proteome composition in winter wheat cultivars (Vítámvás et al., 2012) showed that cold acclimation is an active energy-demanding process accompanied by profound changes in carbohydrate metabolism and ROS-scavenging enzymatic system, increased biosynthesis of methionine and *S*-adenosylmethionine, chaperones, enzymes involved in protein turnover and stress-responsive proteins. Molecular mechanisms helping a cold-sensitive spring wheat cultivar to survive long term exposure to suboptimal temperatures were also investigated from the proteomics point of view (Rinalducci et al., 2011b). Quantitative differences in protein abundance indicated reinforcement in ascorbate recycling, protein processing, and accumulation of the enzyme involved in tetrapyrrole resynthesis, whereas key Krebs cycle enzymes and many photosynthesis related proteins were down-regulated (**Figure 1**). A short term exposure of spring wheat plants to freezing stress caused extensive changes in major metabolic processes with enhanced accumulation of key proteins involved in ROS detoxification, signal transduction, stress and disease resistance (Han et al., 2013).

Recent plant proteomics studies have also been focused on heavy metal stress responses, biochemical mechanisms involved in cellular detoxification and heavy metal tolerance (Li et al., 2013b; Kang et al., 2015). The study of copper-induced stress responses reflected in proteomes of leaves and roots from young common wheat seedlings identified significant enhancement in the abundance of proteins involved in signal transduction, stress defense, and energy production, whereas carbohydrate, protein and photosynthetic metabolisms were severely reduced (Li et al., 2013b). Under copper stress conditions, exogenous jasmonic acid application had a protective effect, and activated transcription of glutathione-*S*-transferase. Organ-specific differences in adaptation to high-level mercury (Hg) stress were revealed for majority of proteins with differentially altered abundance in wheat seedling roots and leaves (Kang et al., 2015). The identified Hg-responsive proteins were associated with the main biological processes (**Figure 1**). Furthermore, application of exogenous ABA facilitated protection against Hg stress identifying potential network of key interacting proteins. These studies provide new insights and will lead to better understanding of heavy-metal stress responses in crop plants.

## Concluding Remarks and Future Challenges

Proteomic data that are particularly informative include quantitative protein profiles, profiles of regulatory modifications and protein interaction networks. Gel-based 2-DE proteomic approaches combined with gel-free MS-based quantitative proteomic techniques have been widely used for crop proteome analysis. The complex mixtures of proteins with the dynamic range of protein concentrations in plant cells have been analyzed more in-depth using a combination of separation techniques based on subcellular proteomics in different stress-responding organs and tissues. Improved protein extraction protocols, advanced gel-free quantitative techniques and bioinformatics approaches to the identification and analysis of complex proteomes at both subcellular and whole plant proteome levels in different crops have significantly advanced our understanding of developmental process, abiotic stress sensing mechanisms, and intracellular signal transduction mechanisms. The recent proteomic studies have contributed to elucidation of complex relationship between stress tolerance and crop productivity, which would enable the development of novel breeding strategies resulting in an increase in crop productivity and environmental performance.

**FIGURE 2:**
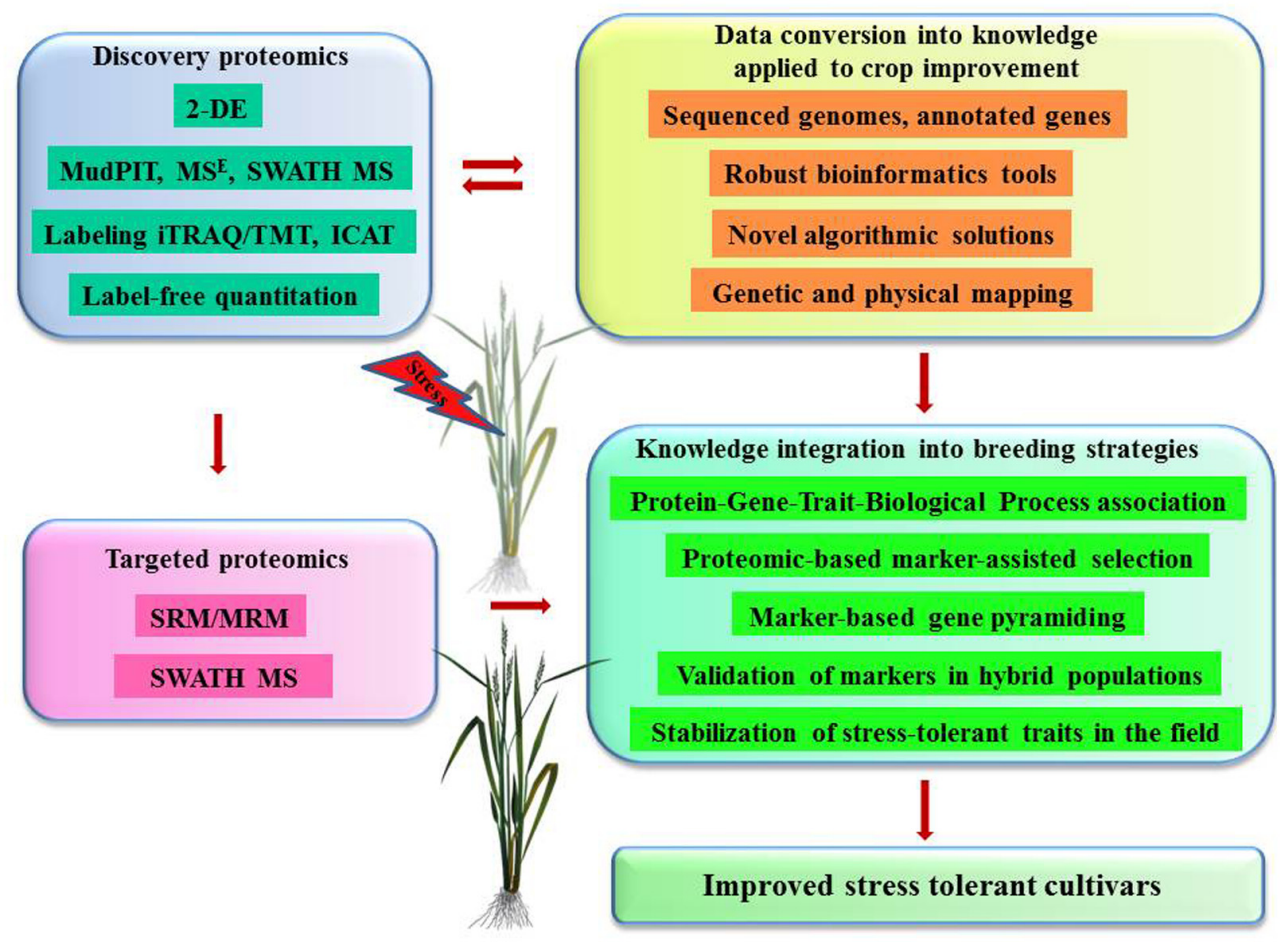
**Proteomics contribution to biotechnology of crop improvement – future challenges and prospects.** Discovery-based proteomics strategies contribute with important information on candidate proteins, biological processes, and mechanisms controlling tolerance to abiotic stresses. This information is further converted into specific knowledge on OTLs, genes, alleles, and their physical chromosomal locations using modern mathematical algorithms and bioinformatics tools. Targeted proteomics strategies facilitate the deployment of knowledge linking genotype and its functionality into breeding programs.

Proteomics is rapidly becoming an indispensable tool for global phenotypic characterization at the molecular level that provides invaluable information about novel gene identifications, role of PTMs and protein interactions, linking genotype and its functionality. This type of information is especially useful in breeding programs offering specific identifications of potential biomarkers for isolation of candidate genes that can be integrated through proteomic-based marker-assisted selection and marker-based gene pyramiding (**Figure 2**). Furthermore, proteomics programs contribute to the analysis of advanced mapping populations such as hybrid doubled haploid lines, near isogenic lines and recombinant inbred lines, which further verify the correlations between responsive quantitative trait loci and stress tolerant phenotypes. One of the challenges is to convert massive data into knowledge that can be readily applied into crop improvement programs. The solution can be found in interdisciplinary approaches, creating sufficient genetic resources, robust bioinformatics tools with novel algorithmic solutions.

## Conflict of Interest Statement

The authors declare that the research was conducted in the absence of any commercial or financial relationships that could be construed as a potential conflict of interest.
